# Utility of neutrophil gelatinase-associated lipocalin in identifying septic cavitary effusions in dogs

**DOI:** 10.1093/jvimsj/aalaf083

**Published:** 2026-02-11

**Authors:** Rachel Meyer, Erin McQuinn, Amanda Kreuder, Alan Hassall, Jean-Sebastien Palerme

**Affiliations:** MedVet Chicago, Chicago, IL 60618, United States; Department of Veterinary Clinical Sciences, College of Veterinary Medicine, Iowa State University, Ames, IA 50011, United States; Department of Veterinary Microbiology and Preventative Medicine, College of Veterinary Medicine, Iowa State University, Ames, IA 50011, United States; Department of Veterinary Microbiology and Preventative Medicine, College of Veterinary Medicine, Iowa State University, Ames, IA 50011, United States; Department of Veterinary Clinical Sciences, College of Veterinary Medicine, Iowa State University, Ames, IA 50011, United States

**Keywords:** biomarker, sepsis, septic peritonitis, cavitary effusion

## Abstract

**Background:**

Neutrophil gelatinase-associated lipocalin (NGAL) is an accurate marker of septic cavitary effusions in people.

**Hypothesis/Objectives:**

To evaluate the utility of serum and effusion NGAL concentrations in differentiating septic effusions from effusions caused by other etiologies in dogs.

**Animals:**

Fifty dogs with pleural or peritoneal effusion.

**Methods:**

Ten dogs were prospectively enrolled into each of 5 groups based on effusion etiology: hypoalbuminemia, increased hydrostatic pressure, neoplastic, inflammatory, and septic. Concentrations of NGAL were measured in both serum and effusion.

**Results:**

While median serum NGAL concentrations did not significantly differ between dogs with hypoalbuminemia (24.8 ng/mL, range 5.0–110.0 ng/mL), increased hydrostatic pressure (13.2 ng/mL, range 5.8–46.9 ng/mL), abdominal neoplasia (13.8 ng/mL, range 3.2–27.3 ng/mL), inflammatory (15.8 ng/mL, 5.6–36.6 ng/mL), or septic causes (19.2 ng/mL, range 7.2–64.8 ng/mL) of effusion (*P* = .272), median effusion NGAL concentrations were significantly higher in the septic group (194.4 ng/mL, range 120.0–1471.1ng/mL) than in the hypoalbuminemic (10.7 ng/mL, range 4.1–27.8 ng/mL, *P* < .001), hydrostatic (22.7 ng/mL, range 11.3–56.7 ng/mL, *P* < .001), neoplastic (65 ng/mL, range 15.7–215.3 ng/mL, *P* < .001), or inflammatory (45 ng/mL, range 33.8–195 ng/mL, *P* < .001) groups.

**Conclusions and clinical importance:**

Concentrations of NGAL in effusions were significantly higher in septic effusions than in effusions of other etiologies. These findings suggest that effusion NGAL concentrations could be a helpful marker in the identification of cases with septic effusion.

## Introduction

Septic peritonitis is a surgical emergency, with mortality rates of 36%–67% in animals.^1^ In human medicine, timely empiric antibiotic administration to septic patients increases survival.^2^ Based on this, early antibiotic and surgical intervention after stabilization remains the standard of care in veterinary medicine.^3^

The gold standard for diagnosing septic effusions remains bacterial culture.^4^ However, this requires specialized equipment and training, and final results are often substantially delayed.^4^ Cytology can provide high specificity and allow for a more rapid diagnosis, but the sensitivity of this technique in dogs and cats remains below 50%.^5^ Therefore, there is interest in point-of-care (POC) biomarkers for rapid diagnosis of septic effusions. Blood-to-effusion lactate and glucose ratios are commonly used in dogs and cats, but their diagnostic performance is inconsistent.^1,4,6–9^ Other biomarkers, including cell-free DNA, nucleosomes, N- terminal pro-C-type natriuretic peptide, procalcitonin, CCL2, CXCL8, IL-10, IL-6, KC-Like, have been evaluated in dogs, but these biomarkers do not reliably distinguish septic from non-septic processes.^1,10^ More direct detection of bacterial components using POC tests have shown promise but still appear to have limited specificity.^11,12^

Neutrophil gelatinase-associated lipocalin (NGAL), a 25 kDa molecule belonging to the lipocalin family, is released from neutrophils, epithelial tissues (kidney, lung, colon, liver, etc.), and some tumor types.^13^ It is freely filtered at the glomerulus and reabsorbed in the proximal tubule, such that only low levels are detectable in the urine under physiologic conditions.^13^ Because of these characteristics, NGAL has been used as a sensitive biomarker of acute kidney injury (AKI) in dogs.^14–20^

NGAL has also been evaluated as a biomarker of septic processes.^13^ Both urinary and serum NGAL concentrations are significantly higher in septic than in non-septic human patients.^14^ Dogs with septic peritonitis have higher preoperative plasma and urinary NGAL levels when compared to control dogs undergoing surgery for intervertebral disc disease.^21^ In horses with septic arthritis, NGAL synovial concentrations are significantly higher when compared to controls and discriminate septic from non-septic cases with a high accuracy.^22^ Peritoneal fluid concentrations of NGAL are also used as a marker of peritonitis, a common complication in peritoneal dialysis, being shown to be an independent predictor of development of peritonitis as well as a negative prognostic marker.^23,24^ NGAL concentrations in pleural effusions from human patients with parapneumonic effusions are higher than in patients with non-infectious effusions.^25^

The aim of this study was to evaluate NGAL concentrations in serum and cavitary fluids from dogs with pleural or peritoneal effusions of various causes, including sepsis. We hypothesized that NGAL levels would be higher in septic than in non-septic effusions and would reliably distinguish septic from non-septic cases.

## Materials and Methods

### Study group

Dogs with pleural or peritoneal effusion identified via ultrasonographic examination presenting to the Lloyd Veterinary Medical Center of Iowa State University were prospectively enrolled in the study. All enrolled dogs were required to have a clinical diagnosis, serum chemistry, effusion cytology, and analysis, as well as bacterial culture of effusion performed within 24 h of admission to the hospital. Serum and effusion samples for NGAL quantification were also obtained at enrollment. The research study was approved by our institutional animal care and use committee (IACUC) and owner consent was obtained for all dogs prior to enrollment.

Effusions were classified based on total nucleated cell count (TNCC) and total protein (TP) according to published guidelines.^26^ Exudates were classified as having TNCC > 3000/μL and TP > 2.5 g/dL, high-protein transudates were classified as having TNCC < 3000/μL and TP > 2.5 g/dL, and low protein-transudates were classified as having TNCC < 3000/μL and TP < 2.5 g/dL.

Based on effusion analysis, clinical characteristics, and clinical diagnosis, dogs were categorized into 1 of 5 groups based on the etiology of effusion: septic, inflammatory, neoplastic, increased hydrostatic pressure, or hypoalbuminemia. Dogs with exudative effusions and either intracytoplasmic bacteria identified on fluid cytology (1/10), a positive bacterial culture of the fluid (0/10), or both (9/10) were classified as septic. Dogs were classified in the inflammatory group if they had an exudative effusion with no bacterial growth on culture and no intracytoplasmic bacteria noted on fluid cytology. Cases with malignant cells identified on effusion cytology were categorized as neoplastic, regardless of TNCC or TP. Dogs with effusions that had fluid analysis consistent with either a low or high-protein transudate in combination with an identified cause of pre-hepatic, hepatic, or post-hepatic portal hypertension or echocardiographic evidence of right-sided heart failure were classified in the increased hydrostatic pressure group. Cases with cavitary fluids caused by hypoalbuminemia were classified as such based on the effusion being a low-protein transudate and a serum albumin < 2.0 g/dL. In order to rule out a concurrent septic component to the effusions, dogs in the neoplastic, increased hydrostatic pressure, and hypoalbuminemic groups also were also required to have no bacterial growth on culture and no intracytoplasmic bacteria noted on fluid cytology. Fulfillment of systemic inflammatory response syndrome (SIRS) criteria was retrospectively assessed in all dogs based on evaluation at presentation (Table 1). Dogs fulfilling 2 or more criteria were considered to have SIRS.^21^

**Table 1:** Systemic inflammatory response syndrome criteria.

**Variable**	**SIRS criteria**
**Heart rate**	>120 beats/min
**Respiratory rate**	>20 breaths/min
**Body temperature**	<38.1 °C or >39.2 °C
**White blood cell count**	<6 × 10^3^ cells/μL or >16 × 10^3^ cells/μL
**Increased band count with normal segmented neutrophil count**	>3%

Dogs were classified as having SIRS if 2 or more criteria were met.

### NGAL quantification

For NGAL quantification, clotted blood samples and effusion samples were centrifuged at 1500 *g* for 10 min and supernatants were frozen at −80°C for later batch analysis. NGAL concentrations were quantified using a canine-specific NGAL ELISA (ALPCO, Salem, USA) according to manufacturer’s instructions with absorbance read at 450 nm with a reference wavelength of 650 nm using a Spectramax 190 plate reader (Molecular Devices, Sunnyvale, USA). For each plate, a standard curve consisting of the following standard concentrations (400, 200, 100, 40, 20, 10, 4, and 0 pg/mL) provided by the manufacturer were tested in duplicate and utilized to determine sample concentrations. Sample concentrations were calculated from ELISA absorbances using a 4-parameter logistic curve fitting (Softmax Pro 3.1.1, Molecular Devices, Sunnyvale, USA). The resulting mean, standard deviation, and coefficient of variation (CV) was provided by the software for each sample, and only results that fell within the range of the standard curve were utilized for further statistical analysis. Samples were initially diluted 1:100 with the manufacturer’s diluent and run in duplicate. Out-of-range samples were re-assayed at higher dilutions as appropriate until results fell within the standard curve. Forty-three of the 50 serum samples tested were able to be measured using the standard 1:100 dilution for this assay; the remaining seven required 1:1000 dilution to test within the standard curve. Of the 50 effusion samples, 24 required additional dilution to test within the standard curve due to significantly higher NGAL concentrations (20 at 1:1000; 3 at 1:10 000; 1 at 1:100 000). To assess the intra-assay variability of the test, the mean CV was calculated as 4.4% for the serum NGAL measurement between duplicates (range 0.2%–17%; median 3.7%) For effusion samples, the average CV was calculated as 6.9% between duplicates (range 0.0%–41.3%; median 4.6%).

As the kit was validated for canine serum and urine but not effusion samples, the manufacturer was consulted and recommended performing a linearity and matrix effect validation for the effusion samples. For the linearity validation, NGAL positive control was to be added in decreasing concentration (200, 100, 50, 25, 12, and 6.25 pg/mL) to an undiluted dog effusion sample believed to be negative for NGAL and measured in duplicate similar to all other samples. Several attempts were made to perform this validation, however, given that all effusion samples collected in this study measured >3000 pg/mL, and that effusion is not a body fluid that can be obtained from normal healthy animals in measurable quantities, we were unable to identify a suitable sample free of NGAL to complete the full linearity validation using the available standards.

To perform the matrix validation, an effusion sample anticipated to have low measured NGAL was selected from the hypoalbuminemic samples and utilized in a set of two dilution series to determine if higher levels of the matrix affected the results. One dilution set had 20 pg NGAL from the manufacturer provided standards (50 μL of 400 pg/mL standard) added to each well of the diluted sample (1:50, 1:100, 1:500, 1:1000, 1:5000, 1:10 000, 1:50 000, and 1:100 000), equivalent to adding 200 pg/mL to each assay; the other dilution set did not contain added NGAL dog standard. All dilutions were tested in duplicate, and no significant matrix effect was observed with the average difference between all dilutions being 177.0 pg/mL (range: 148.7–191.3 pg/mL).

### Statistical analysis

Statistical analysis was performed using a commercial software (R version 4.3.3, http://cran.r-project.org/). Differences for variables between groups were determined using a one-way ANOVA test. Normality of the outcome variables was assessed using the Shapiro–Wilk test for each group across all measured outcomes. For most outcomes, all five groups met the normality assumption (*P* > .05); however, for two outcomes (NGAL effusion concentration and creatinine concentration), one group (inflammatory) showed a mild deviation from normality (*P* < .05). However, residual diagnostics from the one-way ANOVA, including Q–Q plots and residuals vs. fitted value plots, did not reveal substantial departures from normality or indicate heteroscedasticity. Additionally, log-transformed outcome variables were also evaluated and confirmed to meet the assumptions of normality and homogeneity of variance for one-way ANOVA. When variables were significantly different between groups, a pairwise comparison with Tukey’s adjustment was performed. Correlations between NGAL concentrations and effusion cell counts were assessed using Pearson’s product–moment correlation. For binary comparison of values of the septic groups with all other groups taken together, a two-sample t-test was employed with normality being confirmed using a Shapiro-Wilcoxon test. Results were considered statistically significant if *P* < .05. Descriptive statistics including the average, median, min and max CV were calculated in Microsoft Excel (Version 16.89).

## Results

### Study group

Sixty-two dogs were initially enrolled. Twelve dogs were excluded due to incomplete data or ambiguous effusion classification, leaving 50 dogs in the final analysis. The cohort of dogs included in the study was composed of 30 different breeds with the three most common breeds being mixed breeds (9), Labrador retrievers (6), and English bulldogs (3). Twenty-two dogs had pleural effusion sampled and 28 had peritoneal effusion sampled. If bicavitary effusion was present, only 1 effusion source was included per dog. There were 10 dogs in each of the 5 etiologic groups: septic, inflammatory, neoplastic, hydrostatic, and hypoalbuminemic. Twenty-eight were male (23 neutered, 5 intact) and 22 were female (19 spayed, 3 intact). The mean weight was 22.4 kg, with no significant differences among the groups, and the mean age was 7.0 years (range 0.25–16). The septic group (mean 4.3 years) was significantly younger than the inflammatory group (mean 8.9 years; *P* = .0461) and the neoplastic group (9.6 years; *P* = .0461). Evaluation of SIRS criteria revealed that 39/50 dogs in our study fulfilled at least 2 criteria (Table 2).

**Table 2:** Selected mean hematological values, biochemical values, and SIRS criteria fulfillment of dogs with cavitary effusions based on etiology.

**Effusion group**	**Neutrophils count ± SD (×10** ^**3**^ **/μL)**	**BUN ± SD (mg/dL)**	**Creatinine ± SD (mg/dL)**	**Albumin ± SD (g/dL)**	**SIRS cases (/10)**
**Oncotic**	12.9 ± 5.0	14.5 ± 5.4	0.7 ± 0.2	1.6 ± 0.4	9
**Hydrostatic**	9.6 ± 3.3	18.0 ± 10.3	1.0 ± 0.4	3.2 ± 0.5	7
**Neoplastic**	9.2 ± 3.5	13.2 ± 4.3	0.8 ± 0.3	3.1 ± 0.4	7
**Inflammatory**	14.1 ± 8.9	25.1 ± 19.4	1.3 ± 1.1	2.9 ± 0.5	7
**Septic**	10.9 ± 7.7	20.1 ± 17.3	0.7 ± 0.3	2.4 ± 0.6	9

Abbreviations: BUN = blood urea nitrogen; SD = standard deviation; SIRS = systemic inflammatory response syndrome.

### Clinical pathology

Serum biochemical data were available for 50/50 cases (Table 2). Serum albumin was significantly lower in the hypoalbuminemic group (mean 1.6 g/dL ± 0.4) than all other groups (*P* < .001). In addition, the septic group had a significantly lower albumin than the hydrostatic and neoplastic groups (*P* < .001). Serum creatinine and BUN concentrations did not differ significantly between groups (*P* = .18 and *P* = .75, respectively). Hematological data were also available for 46 dogs (Table 2). Neutrophil counts did not differ significantly between groups (*P* = .39).

### Fluid analysis and cytology

Values for TNCC and TP as well as effusion locations for all groups are included in Table 3. All groups differed significantly based on TNCC (*P* < .001) with the exception of the inflammatory group, which had no significant difference with the septic (*P* = .12) and neoplastic groups (*P* = .15). The TP of the hypoalbuminemic group was significantly lower when compared to the 4 other groups (*P* < .05). Cytology of neoplastic cases revealed either atypical epithelial cells (7/10) or intermediate-large lymphocytes (3/10) consistent with a diagnosis of carcinoma or lymphoma, respectively. Concurrent mild to severe neutrophilic or mixed inflammation was reported in 8/10 neoplastic samples.

**Table 3:** Effusion locations and characteristics of dogs with cavitary effusions based on etiology.

**Effusion group**	**Effusion location (pleural/peritoneal)**	**Mean ± SD TNCC (cells/μL)**	**Mean ± SD TP (g/dL)**
**Oncotic**	3/7	248 ± 214	2.38 ± 0.38
**Hydrostatic**	3/7	1035 ± 674	3.51 ± 1.05
**Neoplastic**	7/3	17 363 ± 11 383	3.46 ± 0.68
**Inflammatory**	3/7	38 671 ± 38 942	4.37 ± 0.98
**Septic**	6/4	89 653 ± 70 813	4.03 ± 0.75

Abbreviations: SD = standard deviation; TNCC = total nucleated cell count; TP = total protein.

### NGAL analysis

**Figure 1:**
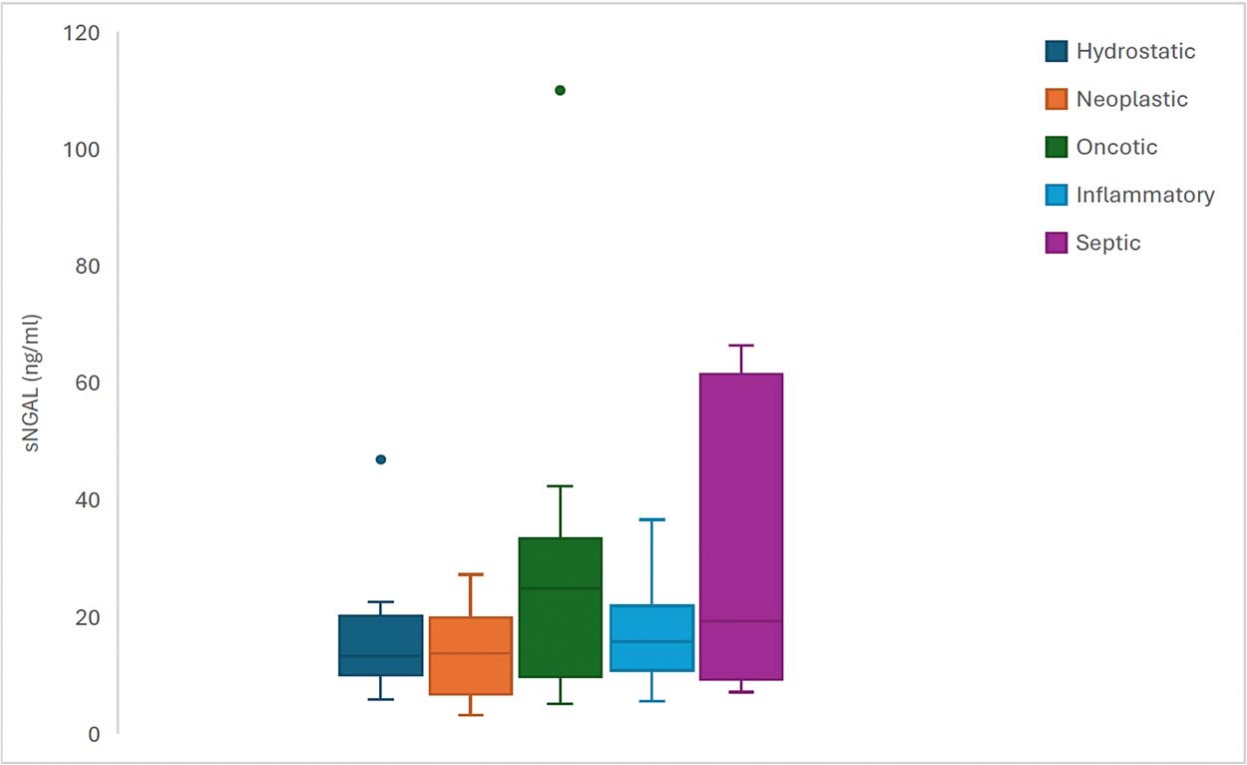
Box and whisker plots of serum NGAL (sNGAL) concentrations in dogs with different etiologies of cavitary effusion. Boxes represent the interquartile range from the 25th to 75th percentiles with horizontal lines representing median values, and T-bars representing minimum and maximum values. Closed circles represent outlying data points.

Serum NGAL concentrations did not differ significantly between hypoalbuminemic (median 24.8 ng/mL, range 5.0–110.0 ng/mL), hydrostatic (median 13.2 ng/mL, range 5.8–46.9 ng/mL), neoplastic (median 13.8 ng/mL, range 3.2–27.3 ng/mL), inflammatory (median 15.8 ng/mL, 5.6–36.6 ng/mL), and septic (median 19.2 ng/mL, range 7.2–64.8 ng/mL) groups (*P* = .27) (Figure 1). The lack of significant difference persisted even when the septic group was compared to all other groups combined (median 15.7 ng/mL, range 3.2–110.0 ng/mL; *P* = .23). Serum NGAL concentrations did not significantly correlate with peripheral blood neutrophil counts (*r* = −0.10, *P* = .51).

**Figure 2:**
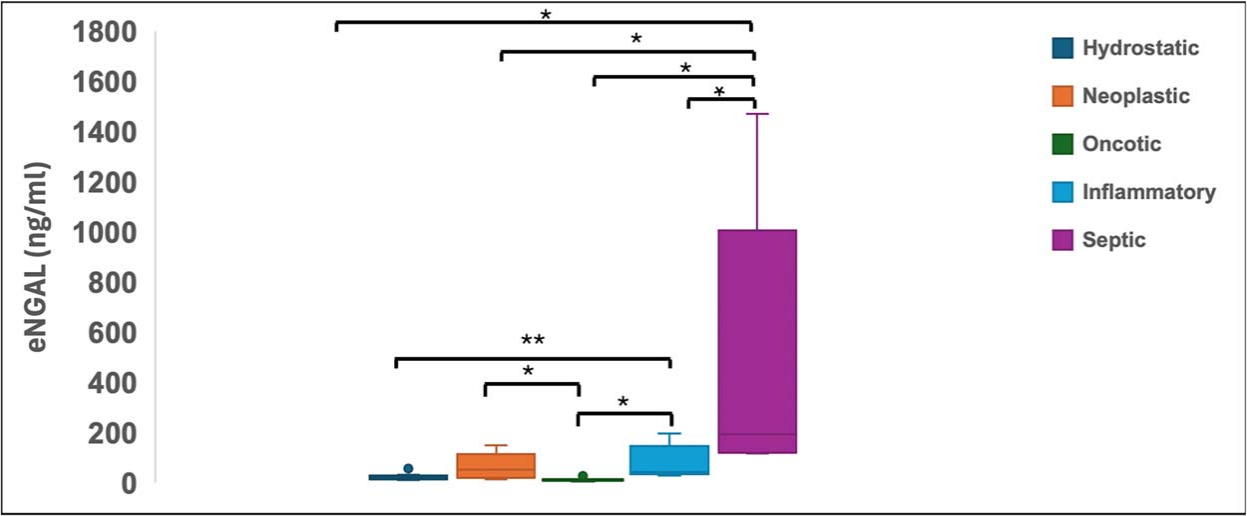
Box and whisker plot of effusion NGAL (eNGAL) concentrations in dogs with different etiologies of cavitary effusion. Boxes represent the interquartile range from the 25th to 75th percentiles with horizontal lines representing median values, and T-bars representing minimum and maximum values. Closed circles represent outlying data points. ^*^P < .01, ^**^P < .05.

Effusion concentrations of NGAL were significantly higher in septic effusions (median 194.4 ng/mL, range 120.0–1471.1 ng/mL) than in the hypoalbuminemic (median 10.7 ng/mL, range 4.1–27.8 ng/mL, *P* < .001), hydrostatic (median 22.7 ng/mL, range 11.3–56.7 ng/mL, *P* < .001), neoplastic (median 65 ng/mL, range 15.7–215.3 ng/mL, *P* < .001), or inflammatory (median 45 ng/mL, range 33.8–195 ng/mL, *P* < .001) groups (Figure 2). The NGAL concentrations of the septic group were still significantly higher when compared to all other groups combined (median 28.0 ng/mL, *P* < .001). Effusion concentrations of NGAL were found to have a moderate positive correlation with effusion TNCC (R = 0.52, *P* < .001).

## Discussion

This study reports the concentrations of NGAL found in serum and cavitary fluids of dogs with effusive diseases of various etiologies and identifies this biomarker as a potentially useful tool for the distinction of septic effusions from effusions of other etiologies.

In contrast to effusion NGAL concentrations, serum NGAL concentrations did not differ significantly between effusion groups in our study. This is an unexpected finding, since studies in both people and animals demonstrated higher NGAL values in plasma or serum from septic patients when compared to healthy controls.^13,21,27–29^ One possible explanation for the lack of significant difference in serum NGAL is the timing of sample collection, as data in horses shows that NGAL concentrations vary at different time points based on the location of the sampled fluid. Indeed, in an LPS-induced model of articular inflammation, synovial NGAL values peaked rapidly at 8 h, while serum NGAL values did not peak until 36 h after injection.^30^ Therefore, it is possible that NGAL might increase in effusions or at the site of infection much earlier than in serum or plasma. Since the time of onset of the septic processes was not known in our study group and since we only collected data at one time point, we cannot discount that sample timing played a role in our finding of a lack of difference in serum NGAL concentrations between groups.

Another confounding dog variable that might have influenced serum NGAL concentrations is the difference in severity of illness in our study cohort. People with sepsis and circulatory shock have higher serum and urinary NGAL concentrations than those without shock, independent of the presence of an AKI.^30^ Septic dogs have significantly higher serum NGAL concentrations than healthy controls.^21^ However, in that study, recruitment was not only based on the presence of septic peritonitis but also the fulfillment of SIRS criteria. This is in contrast to our study, where dogs were recruited solely based on the presence of septic effusion, regardless of SIRS criteria. However, retrospective assessment of SIRS criteria in our study cohort revealed that the majority of dogs of all groups fulfilled at least 2 SIRS criteria. This high rate of SIRS criteria fulfillment in our study cohort underlines the lack of specificity of these criteria as some of them, namely respiratory rates above 20 breaths per minute, are presumably present in most dogs presenting to an ICU. Consequently, it is possible a variety of disease conditions result in higher serum NGAL concentrations, thus decreasing this biomarker’s value in discriminating between critically ill patients. Finally, since the presence of cavitary effusion was an inclusion criterion for our study, we did not include a healthy control group in our study. This leaves open the possibility that while no difference existed in serum NGAL concentrations between dogs with various causes of effusion, their serum NGAL concentrations were higher when compared to healthy dogs. Though previously published serum NGAL concentrations in healthy dogs are not substantially different from those found in our study cohort, a statistical comparison cannot be made.^17,31^

The NGAL concentrations in effusion samples in the present study were approximately 10-fold higher than previously published serum values. However, these values are similar to those reported in people with parapneumonic effusions as well as people developing septic peritonitis secondary to peritoneal dialysis.^24,25^ This substantial difference in NGAL concentrations between compartments could be explained by kinetics of NGAL production as well as by differences in neutrophil concentrations between blood and other compartments. In mice, the neutrophil transcriptional profile changes as they move through different body or fluid compartments.^32^ When peritonitis is induced in mice, one gene that is preferentially increased in effusion compared to blood is *LCN2*, which encodes NGAL. It is therefore possible that NGAL is upregulated earlier following neutrophil extravasation to sites of inflammation and might be higher in effusion relative to blood. Also, since neutrophils are the main source of NGAL production, NGAL concentrations are likely proportional to neutrophil concentrations. Similar to NGAL concentrations, effusion cell counts were almost 10-fold higher than those from peripheral blood samples. Furthermore, we reported a positive correlation between TNCC and NGAL concentrations in effusion but a lack of correlation between serum NGAL concentrations and peripheral blood neutrophil counts. NGAL concentrations differ between fluid compartments and effusion concentrations are orders of magnitude higher than serum concentrations in humans and horses with septic arthritis. Similar to our study, positive correlations with synovial cell counts are reported.^22,33^

Our study includes some important limitations which should be considered when interpreting our findings. First and foremost, the small sample size and absence of a prior power calculation limits our study’s ability to reliably detect true differences between groups, increasing the risk of Type II error. Consequently, it is possible that differences in either serum or effusion NGAL concentrations, or both, did exist between groups other than the septic group. Type I errors are also possible in this context, causing us to falsely identify a difference between groups. However, Type I errors are less common, and our positive findings are supported by the fact the significant difference in effusion NGAL concentration was still present when the septic group was compared to all other dogs taken together as a single group.

For measurement of both serum and effusion NGAL concentrations, we used a commercially available ELISA kit that has been validated for use in canine serum and urine but not cavitary effusions. To address this limitation, we performed a matrix validation to confirm that the assay was able to reliably detect and quantify NGAL in effusion samples. Although we were unable to complete a formal linearity validation due to sample volume constraints, the matrix validation results support the assay’s suitability for this application. In addition, the use of ELISA kits validated for one biological matrix in other, unvalidated matrices is well-established in the veterinary literature. For example, a similar NGAL kit from the same manufacturer—validated only for equine serum—has been successfully applied in published studies measuring NGAL concentrations in equine peritoneal and synovial fluids.^22,34,35^ This precedent, combined with our own matrix validation, supports the appropriateness of using the present kit for canine effusion samples in this exploratory study.

The classification of cases into the neoplastic group in our study was based solely on cytological review of the effusion, not histopathological evaluation, raising the possibility of an incorrect diagnosis. Though cytological evaluation is typically sufficient for diagnosis of round cell neoplasia such as lymphoma, this is a special concern when dealing with carcinomas. Indeed, carcinomas can have overlapping cytological features with non-neoplastic epithelial cells that exfoliate into inflammatory environments, making a definitive diagnosis challenging at times.^36,37^ However, this is unlikely to be an important concern in our study cohort as, of the 6 dogs with a diagnosis of an epithelial neoplasia, 5 had concurrent identification of either a miliary lung pattern or mass on diagnostic imaging, supporting the diagnosis of a neoplastic condition. In addition, two of these cases had confirmation of diagnosis based on necropsy findings. In the remaining dog, only low numbers of leukocytes were reported in the effusion, making a metaplasia less likely than a true neoplasia. Effusion cytology has high specificities for detection of neoplasia and a study evaluating the performance of effusion cytology for the diagnosis of ovarian carcinomas in dogs specifically found effusion cytology to be a dependable modality with accuracies over 90%.^38,39^

A final limitation that should also be considered with our data pertains to our case selection. While inclusion in our septic group required a clear confirmation of infection using cytology, bacteriology, or both, the cases which are most challenging in a clinical setting are those that have low or undetectable numbers of bacteria, either due to dilutional factors or previous antibiotic use. How our data applies to this group remains to be determined and future prospective studies should consider this group in evaluation of this biomarker.

In conclusion, NGAL concentrations in effusions were significantly higher in dogs with septic effusions than in dogs with effusions of other etiologies. This biomarker’s high sensitivity for septic effusions suggests its potential utility in providing a timely diagnosis.

## Off-label antimicrobial declaration

Authors declare no off-label use of antimicrobials.

## Institutional animal care and use committee or other approval declaration

Approved by Iowa State University of Scient and Technology, College of Veterinary Medicine, Institutional Animal Care and Use Committee, protocol number 21-103.

## Abbreviations

NGAL: neutrophil gelatinase-associated lipocalin
TNCC: total nucleated cell count
TP: total protein
